# Sociodemographic correlates of perceived physical literacy in Spanish adolescents: results from the EHDLA study

**DOI:** 10.3389/fspor.2025.1601852

**Published:** 2025-07-24

**Authors:** María Mendoza-Muñoz, José Francisco López-Gil, Damián Pereira-Payo, Raquel Pastor-Cisneros

**Affiliations:** ^1^ Department of Communication and Education, Universidad Loyola Andalucía, Sevilla, Spain; ^2^ School of Medicine, Universidad Espíritu Santo, Samborondón, Ecuador; ^3^ Vicerrectoría de Investigación y Postgrado, Universidad de Los Lagos, Osorno, Chile; ^4^ Health, Economy, Motricity and Education (HEME) Research Group, Faculty of Sport Sciences, University of Extremadura, Cáceres, Spain; ^5^ Physical Activity for Education, Performance and Health (PAEPH) Research Group, Faculty of Sport Sciences, University of Extremadura, Cáceres, Spain

**Keywords:** adolescents, correlates, education, perceived physical literacy, Spain

## Abstract

**Background/Objective:**

Perceived physical literacy (PPL) is a crucial factor influencing adolescents’ engagement in physical activity and overall well-being. This study tried to determine the sociodemographic correlates of PPL among adolescents in Spain.

**Methods:**

A total of 1,378 participants [51% girls, median age = 14 years, interquartile range (IQR) 13 to 16] were analyzed. PPL was assessed via the Spanish Perceived Physical Literacy Scale (S-PPLL), with a median score of 33.0 (IQR 30.0 to 37.0). A generalized linear model was carried out to determine the correlates associated with a higher PPL.

**Results:**

The model revealed significant associations between sex, SES, and maternal education with PPL. Compared with boys, girls presented a lower association with PPL [unstandardized beta coefficient [*B*] = −1.24, 95% confidence interval [CI] −1.93 to −0.55, *p* < 0.001]. A higher SES was positively associated with greater PPL (medium SES: *B* = 1.76, 95% CI 0.81 to 2.70, *p* < 0.001; high SES: *B* = 2.34, 95% CI 1.22 to 3.45, *p* < 0.001), in comparison with those with lower SES. Additionally, maternal education level was positively associated with adolescents’ PPL scores, with higher education levels linked to greater PPL (*B* = 1.48, 95% CI 0.41 to 2.55, *p* = 0.007). However, factors such as immigrant status, paternal education, family structure, number of siblings, type of schooling, and area of residence were not significantly associated with PPL (*p* > 0.05).

**Conclusion:**

Sex, SES, and maternal education could influence adolescents’ PPL, highlighting the need for targeted interventions to address disparities and promote PL.

## 1 Introduction

Physical literacy (PL) is a fundamental concept in the promotion of physical activity (PA) across the lifespan, as it not only encompasses the development of motor skills but also influences confidence, motivation and understanding of the importance of movement for overall wellbeing (1). In adolescence, a critical period for the consolidation of healthy habits, PL plays a determining role in the adoption of an active lifestyle, with direct repercussions for physical, mental and social health (2). However, despite its relevance, perceived physical literacy (PPL) varies significantly among adolescents (3, 4), suggesting the influence of various sociodemographic factors on its development.

Beyond the development of motor skills and the promotion of lifelong physical activity, physical literacy (PL) has been increasingly framed within a more holistic and humanistic perspective. As Durden-Myers, Whitehead, and Pot (5) pointed out, PL contributes to human flourishing by enhancing autonomy, self-expression, and a sense of competence. These dimensions are critical during adolescence, a developmental period marked by identity formation and emotional vulnerability. In this broader view, PL becomes a physical or educational construct and a vital component of personal well-being and social development.

Within this framework, physical education (PE) emerges as a key institutional setting for the structured development of PL among children and adolescents. PE classes provide regular, inclusive, and pedagogically guided opportunities to foster not just physical competence, but also motivation, confidence, and knowledge about movement and health (6). Moreover, there is growing consensus around the importance of explicitly assessing and developing PL within school curricula to ensure that all students (regardless of background) may engage meaningfully in physical activity across the life course (7). As such, the educational system plays a central role in ensuring equitable access to PL, particularly for vulnerable groups who may face additional social or structural barriers.

Despite its recognized importance, PPL remains an underexplored area in relation to sociodemographic disparities, particularly in adolescents. However, many studies have examined the determinants of PA, addressing aspects such as sex, socioeconomic status (SES) (8, 9), and parental education (10, 11). Given that PL is not only about motor skill acquisition but also about fostering confidence, motivation, and understanding of movement's role in overall wellbeing (1), it is vital to examine how sociodemographic disparities influence its development.

One of the most consistently reported inequalities in PA is sex-based differences, with studies showing that girls tend to participate less in structured and unstructured PA than boys do (12). However, research on gender disparities in PPL is still limited, despite its crucial role in shaping lifelong engagement in movement. The few existing studies suggest that girls have poorer PPL than boys do (3, 4).

Similarly, SES is a well-established determinant of PA (8), but its impact on PL has received little attention in the literature. Adolescents from lower-income families face significant barriers to developing PA, including limited access to sports facilities, extracurricular programs, and parental support (9).

Parental education has also been linked to adolescent PA levels. Compared with parents with intermediate or higher education levels, parents with lower education levels engage in less total PA (10). Thus, it is important to focus on children and adolescents whose parents have lower educational levels to help them maintain PA during adolescence and to inform them about the benefits of regular PA and its essential role in promoting proper health and growth (11).

Understanding the sociodemographic factors that influence adolescent PL is crucial for developing policies and intervention strategies that promote equitable participation in PA. Inequity in PL can lead to differences in health across the lifespan, contributing to increased sedentary lifestyles and chronic diseases, especially in vulnerable populations (13).

From a public health perspective, identifying groups with lower PL will enable targeted programmes to reduce gaps, ensuring that all adolescents have access to opportunities to develop their motor skills and confidence in PA (14).

Therefore, this study aimed to analyze the associations between different sociodemographic factors and the perception of PL in adolescents, identify which variables have a significant effect on PL, and provide evidence for the design of strategies to promote a more equitable development of PL.

## 2 Material and methods

### 2.1 Study design

A cross-sectional study was carried out to analyze secondary data from a sample of adolescents aged 12–17 years [median = 14.0; interquartile range (IQR) 13.0 to 16.0] from *Valle de Ricote* (Region of Murcia, Spain) enrolled in the Eating Healthy and Daily Life Activities (EHDLA) (15). The original sample consisted of 1,378 adolescents (51% girls) from three secondary schools. The data were collected during the 2021–2022 academic year.

For the students’ participation in the study, the parents or legal guardians of the adolescents received a written information sheet explaining the objectives of the research project and the tests and questionnaires administered, as well as an informed consent form that they had to sign before the enrollment of the participants. The adolescents also had to give their consent to participate in the study.

The following inclusion criteria were considered for the study: (1) being between 12 and 17 years old and (2) being registered and/or residing in the *Valle de Ricote*. In addition, the following exclusion criteria were used: (1) adolescents who were exempt from the subject of physical education at school (since all the measurements in the study were carried out during physical education classes); (2) suffered from a pathology that prevented them from practising PA; (3) were under some type of pharmacological treatment; (4) their parents or legal guardians did not authorize their participation in the study; or (5) did not agree to participate in the study.

### 2.2 Ethics

This study was approved by the Bioethics Committee of the University of Murcia (ID-2218/2018) and the Ethics Committee of the Albacete University Hospital Complex and Albacete Integrated Care Management (ID-2021-85). In addition, we followed the updates of the Declaration of Helsinki and respected the human rights of the registered participants.

### 2.3 Procedures

#### 2.3.1 Perceived physical literacy instrument (PPLI)

The original Perceived Physical Literacy Instrument (PPLI) was initially designed for physical education teachers and comprises 18 items (16). A validated version adapted for adolescents was subsequently developed, consisting of nine items (17). In the present study, the Spanish Perceived Physical Literacy Instrument for Adolescents (S-PPLI) was utilized to assess PL in adolescents (3). The S-PPLI has been previously validated for use with Spanish youth, ensuring its reliability and applicability within this population. This version preserved the nine-item structure, with participants rating each statement on a 5-point Likert scale ranging from 1 (strongly disagree) to 5 (strongly agree). The instrument comprised three key domains (i.e., knowledge and comprehension, self-expression and interaction with others, and self-perception and self-confidence), each containing an equal number of items. Intraclass correlation coefficients (ICC) indicated moderate/good reliability in Spanish adolescents (ICC 0.62–0.79). Moreover, an adequate internal consistency was obtained [McDonald's *ω* = 0.87; 95% confidence interval (CI), 0.81–0.86; Cronbach's *α* = 0.87; 95% CI 0.85–0.89].

#### 2.3.2 Sociodemographic variables

General information on the participants’ sex and age was recorded. The schooling type was classified as either public or private, while the area of residence was categorized into urban (more than 5,000 inhabitants) or rural (5,000 or fewer inhabitants) (18).

The immigrant status of students was determined on the basis of at least one of the following criteria: being a child of immigrant parents, being born outside Spain, or having at least one parent born abroad.

Socioeconomic status (SES) was assessed using the Family Affluence Scale III (FAS-III) (19). The FAS-III includes six items: (a) number of vehicles owned by the family, (b) having one's own bedroom, (c) number of computers in the household, (d) number of bathrooms in the home, (e) presence of a dishwasher, and (f) frequency of family holidays abroad during the past year. Each item is scored individually, and the sum yields a total score ranging from 0 to 13. SES categories were derived based on the internationally established cutoffs used in the Health Behaviour in School-aged Children (HBSC) study: low (0–2 points), medium (3–5 points), and high (≥6 points). Although these cutoffs have not been specifically validated in the Spanish population, their use ensures comparability with international studies and aligns with standard practice in FAS-III–based research. Additionally, participants provided information regarding the educational attainment of their father, mother, or legal guardian, selecting from the following levels: incomplete primary education, complete primary education, incomplete secondary education, complete secondary education, incomplete higher education, or complete higher education.

Furthermore, adolescents were asked about their family environment (including the number of people residing in their home and the number of siblings). They also specified their family structure, choosing among the following categories: To assess the family environment, adolescents were asked about various household characteristics, including the number of people residing in their home and the number of siblings. They also specified their family structure [nuclear family: two biological parents (father and mother) and their children; single-parent family: a single parent (father or mother) responsible for raising the children; extended family: parents and children cohabiting with other relatives (e.g., grandparents, aunts, uncles, cousins); diverse family: nontraditional family structures, including same-sex parents, blended families, foster families, adoptive families, and other nonconventional arrangements).

### 2.4 Statistical analysis

To assess the normality of the variables, visual techniques such as density and quantile‒quantile plots were used, complemented by the Shapiro‒Wilk test. The median with its IQR is shown for quantitative variables, and frequencies (n) and percentages (%) are displayed for qualitative variables. To mitigate the effects of missing data, we employed multiple imputation using chained equations (MICE) through the “*mice*” package. In line with established guidelines, we created 26 imputed datasets, which is more than 100 times the highest percentage of missing data in any variable. This approach enabled us to confirm the robustness of our results and minimize the risk of selection bias from excluding cases with incomplete data. To evaluate the validity of the missing at random (MAR) assumption, we conducted Little's missing completely at random (MCAR) test using the “*mcar_test*” function from the “*naniar*” package. The test results indicated that the data were not missing completely at random (MCAR) [chi-square [*χ*^2^] = 479, degrees of freedom [*df*] = 86, *p* < 0.001]. Visual inspections of descriptive statistics between participants with and without missing data on key variables showed no substantial differences (Supplementary Table S1), supporting the MAR assumption and validating the imputation method. For the primary analysis, we used listwise deletion, including only complete cases in the robust generalized linear model (GLM) with a Gaussian distribution was carried out to verify the sociodemographic factors linked to PPL among the sample of adolescents examined. To ensure robustness against potential violations of assumptions such as heteroscedasticity, non-normal errors, and the presence of outliers, we employed robust regression using the “*lmrob*” function from the “*robustbase*” package. Robust standard errors were computed using asymptotic variance estimates suitable for the “*SMDM*” estimation procedure. The results are presented as the unstandardized beta coefficient (*B*) and 95% CI. As a sensitivity analysis, the GLM was repeated using the imputed data, and the results are reported separately. Statistical analyses were carried out via R software (version 4.3.2), which was developed by the R Core Team in Vienna, Austria, alongside RStudio (version 2023.12.1 + 402) from Posit, which is based in Boston, MA, USA. Statistical significance was defined as a *p* value < 0.05 for all analyses.

## 3 Results

Table 1 presents the descriptive characteristics of the study participants, who were girls (51%). The median age was 14.0 years (IQR 13.0 to 16.0). With respect to SES, 53% of the participants belonged to the medium SES group, whereas 22% and 25% were classified as having low and high SES, respectively. Most participants were native (75%), and 83% identified as Caucasian. In terms of parental education, most mothers (42%) and fathers (42%) had secondary education, whereas 31% of mothers and 36% of fathers had primary education or lower. The family structure was predominantly nuclear (81%), and 74% of the participants resided in urban areas. Additionally, 81% were enrolled in public schools. The median Spanish Perceived Physical Literacy (S-PPLL) score was 33.0 (IQR 30.0 to 37.0).

**Table 1:** Descriptive data of the study participants.

Variable	*N* = 1,378
Age	14.0 (13.0, 16.0)
Sex	
Boys	680 (49%)
Girls	698 (51%)
SES	
Low	243 (22%)
Medium	585 (53%)
High	283 (25%)
Missing	267
Immigrant status	
Native	836 (75%)
Immigrant	275 (25%)
Missing	267
Number of siblings	1.0 (1.0, 2.0)
Missing	267
Number of people at home	3.0 (3.0, 4.0)
Missing	267
Educational level (mother)	
Primary education or lower	342 (31%)
Secondary education	458 (42%)
University education	293 (27%)
Missing	285
Educational level (father)	
Primary education or lower	385 (36%)
Secondary education	443 (42%)
University education	228 (22%)
Missing	322
Race/ethnicity	
Caucasian	924 (83%)
Non-Caucasian	187 (17%)
Missing	267
Type of family	
Nuclear	902 (81%)
Single-parent	73 (6.6%)
Extended	37 (3.3%)
Diverse	99 (8.9%)
Missing	267
Type of schooling	
Public	1,123 (81%)
Private	255 (19%)
Area of residence	
Urban	823 (74%)
Rural	288 (26%)
Missing	267
S-PPLI (score)	33.0 (30.0, 37.0)
Missing	301

Median (interquartile range) or number (%). S-PPLI, Spanish perceived physical literacy; SES, socioeconomic status.

Table 2 presents the results of the generalized linear model analyzing the associations between various sociodemographic covariates and adolescents’ PPL. The model revealed significant associations between sex, SES, and maternal education with PPL. Compared with boys, girls presented a lower association with PPL (*B* = −1.24, 95% CI −1.93 to −0.55, *p* < 0.001). A higher SES was positively associated with greater PPL (medium SES: *B* = 1.76, 95% CI 0.81 to 2.70, *p* < 0.001; high SES: *B* = 2.34, 95% CI = 1.22 to 3.45, *p* < 0.001), in comparison with those with lower SES. Additionally, maternal education level was positively associated with adolescents’ PPL scores, with higher education levels linked to greater PPL (*B* = 1.48, 95% CI 0.41 to 2.55, *p* = 0.007). In contrast, variables such as immigrant status, paternal educational level, family structure, number of siblings, type of schooling, and area of residence did not show significant associations with PPL in the analyzed sample (*p* > 0.05). Results from the GLM using a multiple imputation approach are provided in Supplementary Table S2, yielding consistent estimates with those obtained from the listwise deletion approach.

**Table 2:** Generalized linear model examining the associations between sociodemographic covariates and perceived physical literacy among adolescents (*N* = 1,026).

Predictor	*B*	95% CI	*p* value
Age (per year)	−0.13	−0.35, 0.10	0.265
Sex			
Boys	Reference		
Girls	−1.24	−1.93, −0.55	<0.001
SES			
Low SES	Reference		
Medium SES	1.76	0.81, 2.70	<0.001
High SES	2.34	1.22, 3.45	<0.001
Immigrant status			
Native	Reference		
Immigrant	0.36	−0.76, 1.48	0.530
Number of siblings (per sibling)	−0.06	−0.50, 0.39	0.804
Number of people at home (per person)	−0.06	−0.46, 0.35	0.779
Educational level (mother)			
Primary education or lower	Reference		
Secondary education	0.61	−0.28, 1.49	0.177
University education	1.48	0.41, 2.55	0.007
Educational level (father)			
Primary education or lower	Reference		
Secondary education	−0.25	−1.10, 0.60	0.570
University education	−0.41	−1.49, 0.67	0.458
Race/ethnicity			
Caucasian	Reference		
Non-Caucasian	−1.00	−2.31, 0.30	0.131
Type of family			
Nuclear	Reference		
Single-parent	−0.67	−2.47, 1.12	0.462
Extended	1.17	−1.00, 3.34	0.289
Diverse	0.42	−0.88, 1.71	0.530
Type of schooling			
Public	Reference		
Private with public funds	0.39	−0.50, 1.28	0.393
Area of residence			
Urban	Reference		
Rural	−0.19	−0.98, 0.60	0.640

*B*, unstandardised beta coefficient; CI, confidence interval; SES, socioeconomic status.

## 4 Discussion

The results of this study provide important information on the sociodemographic determinants of PPL in adolescents. Our results indicate that sex, SES, and mothers’ educational level were significant predictors of PPL. Specifically, being a girl was associated with a lower level of PPL compared to being a boy, while adolescents with higher SES and those whose mothers were university educated were associated with higher PPL. These findings contribute to the literature on PL and align with previous research emphasizing the role of sociodemographic factors in shaping behaviors, specifically PA (9, 11, 12).

The observed association between female sex and lower PPL is consistent with previous studies highlighting sex disparities in children and adolescents in PA (12), PL (20, 21), and PPL (3, 4, 22). This discrepancy may be attributed to a variety of factors, such as social and cultural norms that discourage female participation in physical activities, lower levels of self-efficacy in sports, and fewer opportunities for structured physical education tailored to girls’ interests (12). Sex differences in motivation and confidence in PAs have been reported (23), and boys tend to show greater self-perceptions of their physical abilities than girls do (24), also perceiving girls as more barriers (23). Specifically, Corr, McSharry, and Murtagh (25) highlight that adolescent girls often avoid PA because of low perceived competence, fear of embarrassment, and lack of inclusive activities. The school focus on competitive sports could marginalize those with lower ability, further demotivating them, as being a competitive activity seems to be a potential barrier in sport and PA activities (26). Competence in motor skills is key to self-esteem and participation in PA (27). However, the focus on competitive team sports in schools marginalizes those with lower motor competence and may lead them to avoid PAs in which they feel they will succeed (25, 28). In addition, Melby et al. (29) reported more pronounced associations between PL and well-being among girls than among boys, which may place them in a more vulnerable position to develop mental health problems (30).

Considering these disparities, schools (and particularly physical education) have a critical role in mitigating gaps in physical literacy. Physical education is not merely a venue for physical training, but rather a foundational context where adolescents can develop the broader components of PL, including knowledge, confidence, and motivation (6). Structured, inclusive, and student-centered PE programs can provide a supportive environment that fosters meaningful engagement with physical activity, especially for those who might feel alienated by competitive or skill-focused sports models. Therefore, to address these disparities, specific interventions are needed to foster inclusive environments for girls and promote their participation in PL activities from an early age.

On the other hand, higher SES was a significant predictor of greater PPL, with adolescents with medium and high SES showing significantly higher scores on PPL than adolescents with lower SES. This finding is consistent with previous research indicating that socioeconomic disparities influence access to PA resources, including sports facilities, after-school programmes and organized physical education opportunities (8, 9). Adolescents from lower socioeconomic backgrounds often face economic and environmental barriers, such as limited access to safe recreational spaces, less parental support for sports participation and less availability of after-school activities (9). Furthermore (31), reported that family SES, together with parental involvement in youth sports, can significantly and positively predict adolescents’ physical and mental health levels. Therefore, low SES could contribute to lower levels of PA and PL in this population, highlighting the need for community policies and programmes that provide equitable opportunities for all adolescents to participate in PA.

Moreover, incorporating PL more intentionally into PE curricula may help ensure that all students, regardless of sex or socioeconomic background, can benefit from its developmental potential. As highlighted by Young et al. (7), assessment frameworks and teaching strategies that address PL holistically are essential to promote equitable outcomes and identify students who may need additional support. This is particularly relevant given that PL, as a multidimensional construct, is closely linked to adolescent identity, social integration, and emotional well-being.

Another significant finding of this study was the positive association between maternal education level and adolescent PPL. Adolescents whose mothers had a university education reported higher PPL scores than those whose mothers had only primary education or less. Evidence suggests that parents with a higher level of education tend to be better informed and more aware of the importance of PA for the health status of their children (32). Specifically, Karki Nepal (33) reported that a strong and direct relationship exists between mothers’ education and child health. Our results are therefore supported by previous research suggesting that parental education, particularly maternal education, plays a crucial role in shaping children's health behaviors, such as healthy dietary habits, screen time (34), and, in particular, participation in PA (10, 11). Educated mothers are more likely to be aware of the benefits of PL and encourage their children to participate in physical activities through active parenting practices, such as enrolling them in sports programmes, promoting outdoor play and emphasizing the importance of an active lifestyle (11). This could be related to data from Chen et al. (22) indicating that parental support has a positive effect on PL, improving children's sports knowledge, emotional well-being and athletic ability.

These results, in line with those of previous studies (35, 36), highlight the interconnectedness of parental education, SES and adolescent participation in PA and the development of PL. Future interventions should consider these structural factors and seek equitable access to PA opportunities, particularly for families with lower educational and economic resources.

Interestingly, sociodemographic factors, such as immigrant status, parental education, family structure, number of siblings, type of schooling and area of residence, were not significantly associated with PPL in our sample. These results suggest that while certain socioeconomic and parental factors play crucial roles in shaping PPL, other demographic variables may have more complex or indirect influences. For example, the lack of a significant relationship between parental education and PPL contrasts with some previous studies that have related general parental education to PA behaviors (37, 38). Similarly, Glozah and Pevalin (39) reported no significant association between parental education and PA but noted a significant relationship with eating behavior. This discrepancy could be due to the different roles of mothers and fathers in shaping children's health behaviors, or it could indicate that maternal influence is stronger in promoting PL during adolescence. Similarly, the absence of a significant association with immigrant status and area of residence may suggest that other unmeasured factors, such as cultural values or school interventions, mitigate these potential disparities. Also, a possible explanation for this could be the threshold used to categorize rural and urban areas. In our case, the municipalities included in the study are geographically close to each other, which may reduce the differences typically observed in studies using different classification criteria in other countries or geographic regions. In this context, Sterdt, Liersch, and Walter (38) highlighted inconsistent associations between PAs and setting (rural/urban), as did Chen et al. (22), who argued that personalized approaches that consider the challenges and advantages of urban and rural settings are needed. The fact that migration status is not associated with PL could indicate that the immigrant adolescents in the sample have an adequate level of integration into the country's educational and sporting system. Although migration status was measured, the length of residence in the country was not considered, which could be a key factor in understanding its relationship with PL. The integration process varies significantly depending on how long an individual has lived in a new country, with recent immigrants potentially facing more cultural and structural barriers to participating in physical activity and sports than those who have resided in the country for several years. Previous research has suggested that longer residences are associated with greater cultural adaptation and participation in host-country behaviors, including engagement in the educational and sporting system (40, 41). Therefore, the lack of association between migration status and PL in our study may be partly explained by differences in the length of time participants had lived in the country, which we were unable to account for. Future research should consider incorporating duration of residence as a potential. In short, the lack of association of these variables could be explained by the interaction of multiple contextual and cultural factors that mediate the relationship between the sociodemographic environment and PL.

This study provides new insights into the sociodemographic determinants of PPL in adolescents, addressing gaps in the literature by considering a wide range of influencing factors. Unlike prior research, which often examines isolated variables, our analysis incorporates sex, SES, parental education, family structure, and area of residence. The use of a robust GLM ensures a rigorous statistical approach, controlling for heteroscedasticity and outliers, thereby strengthening the reliability of the results. A key strength of this study is the large and diverse sample of adolescents, along with the use of a validated assessment tool (i.e., S-PPLL), allowing for a comprehensive evaluation of PPL disparities.

However, certain limitations should be acknowledged: the cross-sectional design does not allow for causal conclusions, and the reliance on self-reported data may introduce recall or social desirability biases. Additionally, the specific geographic and cultural context may limit the generalizability of the findings. The study was conducted in a predominantly rural area of southeastern Spain (*Valle de Ricote*), where sociocultural factors, school infrastructure, and access to extracurricular physical activity may differ from those in urban or other regional contexts nationally or internationally. These particularities could influence how adolescents perceive and engage with physical literacy.

Furthermore, PA levels were not assessed or controlled for in the present study. Given that PA is closely related to the development and PPL, its absence limits the ability to determine whether sociodemographic factors influence PPL directly or indirectly through PA engagement. Future studies should include objective or self-reported PA measures to better understand these interrelations.

Despite these constraints, this study underscores the importance of sociodemographic factors in shaping PL, highlighting the need for targeted interventions to address disparities. Future longitudinal research is essential to explore causal mechanisms and refine strategies to support equitable PL development in adolescents.

## 5 Conclusions

The findings of this study highlight the potential influence of sociodemographic factors on adolescents’ PPL, emphasizing the need for targeted strategies to reduce disparities. Sex and SES emerged as key determinants, with girls reporting lower PL levels than boys and higher SES being associated with greater perceived PL. Additionally, maternal education showed a positive association, suggesting that higher parental education may contribute to better PL outcomes. In contrast, factors such as immigration status, paternal education, family structure, number of siblings, type of schooling, and area of residence did not show significant associations. These results contribute to a deeper understanding of the sociodemographic influences on PL, reinforcing the importance of policies and interventions that promote equitable access to opportunities for physical development. Future research should adopt longitudinal designs to establish causal relationships and explore additional variables that may shape adolescents’ engagement in physical activities and their overall PL. Additionally, it would be valuable to investigate the effectiveness of school- and community-based interventions aimed at reducing the observed disparities, particularly among girls and adolescents from lower SES. Exploring other potentially influential factors, such as cultural values, school environment, or peer influence, as well as integrating qualitative approaches to understand adolescents’ perceptions of PL, could also provide deeper insight into the development of equitable strategies.

## Data Availability

The raw data supporting the conclusions of this article will be made available by the authors, without undue reservation.
