# Supplementary material for: Sociodemographic correlates of perceived physical literacy in Spanish adolescents: results from the EHDLA study
Source: Front Sports Act Living. 2025 Jul 24;7:1601852. doi: 10.3389/fspor.2025.1601852 (PMC12328388; doi:10.3389/fspor.2025.1601852)
Supplement: Supplementary file 1 [file Table1.docx]

Table S1. Comparison of descriptive data between listwise deletion method and multiple imputation method.

| **Variable** | **Listwise deletion method** | **Multiple imputation method** |
| --- | --- | --- |
| Age | 14.0 (13.0, 16.0) | 14.0 (13.0, 16.0) |
| Sex |  |  |
| Boys | 680 (49.3%) | 680 (49.3%) |
| Girls | 698 (50.7%) | 698 (50.7%) |
| SES status |  |  |
| Low | 243 (21.9%) | 300 (21.8%) |
| Medium | 585 (52.7%) | 721 (52.3%) |
| High | 283 (25.5%) | 357 (25.9%) |
| Missing | 267 | 0 |
| Immigrant status |  |  |
| Native | 836 (75.2%) | 1,038 (75.3%) |
| Immigrant | 275 (24.8%) | 340 (24.7%) |
| Missing | 267 | 0 |
| Number of siblings | 1.0 (1.0, 2.0) | 1.0 (1.0, 2.0) |
| Missing | 267 | 0 |
| Number of people at home | 3.0 (3.0, 4.0) | 3.0 (3.0, 4.0) |
| Missing | 267 | 0 |
| Educational level (mother) |  |  |
| Primary education or lower | 342 (31.3%) | 437 (31.7%) |
| Secondary education | 458 (41.9%) | 571 (41.4%) |
| University education | 293 (26.8%) | 370 (26.9%) |
| Missing | 285 | 0 |
| Educational level (father) |  |  |
| Primary education or lower | 385 (36.5%) | 532 (38.6%) |
| Secondary education | 443 (42.0%) | 549 (39.8%) |
| University education | 228 (21.6%) | 297 (21.6%) |
| Missing | 322 | 0 |
| Race/ethnicity |  |  |
| Caucasian | 924 (83.2%) | 1,144 (83.0%) |
| Non-Caucasian | 187 (16.8%) | 234 (17.0%) |
| Missing | 267 | 0 |
| Type of family |  |  |
| Nuclear | 902 (81.2%) | 1,119 (81.2%) |
| Single-parent | 73 (6.6%) | 95 (6.9%) |
| Extended | 37 (3.3%) | 47 (3.4%) |
| Diverse | 99 (8.9%) | 117 (8.5%) |
| Missing | 267 | 0 |
| Type of schooling |  |  |
| Public | 1,123 (81.5%) | 1,123 (81.5%) |
| Private | 255 (18.5%) | 255 (18.5%) |
| Area of residence |  |  |
| Urban | 823 (74.1%) | 1,012 (73.4%) |
| Rural | 288 (25.9%) | 366 (26.6%) |
| Missing | 267 | 0 |
| S-PPLI (score) | 33.0 (30.0, 37.0) | 33.0 (30.0, 37.0) |
| Missing | 301 | 0 |

Median (interquartile range) or number (%). S-PPLI, Spanish Perceived Physical Literacy; SES, socioeconomic status.
