# Supplementary material for: Sociodemographic correlates of perceived physical literacy in Spanish adolescents: results from the EHDLA study
Source: Front Sports Act Living. 2025 Jul 24;7:1601852. doi: 10.3389/fspor.2025.1601852 (PMC12328388; doi:10.3389/fspor.2025.1601852)
Supplement: Supplementary file 2 [file Table2.docx]

Table S2. Generalized linear model examining the association of sociodemographic covariates and perceived physical literacy among adolescents, using a multiple imputation method.

| **Predictor** | ***B*** | **95% CI** | ***p*-value** |
| --- | --- | --- | --- |
| Age (per year) | -0.13 | -0.31, 0.04 | 0.135 |
| Sex |  |  |  |
| Boys | Reference |  |  |
| Girls | -1.15 | -1.75, -0.56 | <0.001 |
| SES |  |  |  |
| Low SES | Reference |  |  |
| Medium SES | 1.74 | 0.94, 2.55 | <0.001 |
| High SES | 2.41 | 1.45, 3.36 | <0.001 |
| Immigrant status |  |  |  |
| Native | Reference |  |  |
| Immigrant | -0.06 | -0.99, 0.88 | 0.904 |
| Number of siblings (per sibling) | -0.01 | -0.38, 0.36 | 0.966 |
| Number of people at home (per person) | 0.01 | -0.33, 0.35 | 0.957 |
| Educational level (mother) |  |  |  |
| Primary education or lower | Reference |  |  |
| Secondary education | 0.72 | -0.04, 1.47 | 0.062 |
| University education | 1.35 | 0.41, 2.29 | 0.005 |
| Educational level (father) |  |  |  |
| Primary education or lower | Reference |  |  |
| Secondary education | -0.01 | -0.74, 0.72 | 0.976 |
| University education | -0.25 | -1.19, 0.70 | 0.608 |
| Race/ethnicity |  |  |  |
| Caucasian | Reference |  |  |
| Non-Caucasian | -0.99 | -2.07, 0.08 | 0.071 |
| Type of family |  |  |  |
| Nuclear | Reference |  |  |
| Single-parent | -0.32 | -1.54, 0.90 | 0.606 |
| Extended | -0.30 | -1.98, 1.39 | 0.729 |
| Diverse | 0.11 | -0.99, 1.21 | 0.847 |
| Type of schooling | 0.18 | -0.63, 0.98 | 0.669 |
| Area of residence |  |  |  |
| Urban | Reference |  |  |
| Rural | -0.15 | -0.83, 0.53 | 0.667 |
| *B*, unstandardized beta coefficient; CI, confidence interval; SES, socioeconomic status. | | | |
